# Siplizumab Induces NK Cell Fratricide Through Antibody-Dependent Cell-Mediated Cytotoxicity

**DOI:** 10.3389/fimmu.2021.599526

**Published:** 2021-02-11

**Authors:** Christian Binder, Felix Sellberg, Filip Cvetkovski, Stefan Berg, Erik Berglund, David Berglund

**Affiliations:** ^1^ Department of Immunology, Genetics and Pathology, Section of Clinical Immunology, Uppsala University, Uppsala, Sweden; ^2^ Research and Development, ITB-Med AB, Stockholm, Sweden; ^3^ Division of Transplantation Surgery, Department of Clinical Science, Intervention and Technology (CLINTEC), Karolinska Institute and Karolinska University Hospital, Stockholm, Sweden

**Keywords:** NK cell, CD2, siplizumab, spontaneous cytotoxicity, antibody-dependent cell-mediated cytotoxicity, NK alloreactivity

## Abstract

The glycoprotein CD2 is expressed on T and NK cells and contributes to cell-cell conjugation, agonistic signaling and actin cytoskeleton rearrangement. CD2 has previously been shown to have an important function in natural NK cell cytotoxicity but to be expendable in antibody-mediated cytotoxicity. Siplizumab is a monoclonal anti-CD2 IgG1 antibody that is currently undergoing clinical trials in the field of transplantation. This study investigated the effect of CD2 binding and Fc γ receptor binding by siplizumab (Fc-active) and Fc-silent anti-CD2 monoclonal antibodies in allogeneic mixed lymphocyte reaction and autologous lymphocyte culture. Further, induction of NK cell fratricide and inhibition of natural cytotoxicity as well as antibody-dependent cytotoxicity by these agents were assessed. Blockade of CD2 *via* monoclonal antibodies in the absence of Fc γ receptor binding inhibited NK cell activation in allogeneic mixed lymphocyte reaction. In contrast, siplizumab increased NK cell activation in both mixed lymphocyte reaction and autologous lymphocyte culture due to FcγRIIIA binding. However, experiments using purified NK cells did not show an inhibitory effect of CD2 blockade on natural cytotoxicity or antibody-dependent cytotoxicity. Lastly, it was shown that siplizumab induces NK cell fratricide. Concluding, siplizumab is a promising biopharmaceutical drug candidate for depletion of T and NK cells with minimal off-target effects.

## Introduction

The glycoprotein CD2 (also known as LFA2) is expressed on T and NK cells where it serves as an adhesion and activation receptor (1). In humans, the main binding partner of CD2 is lymphocyte associated antigen 3 (LFA3; also known as CD58) which is broadly expressed, especially on antigen-presenting cells (APCs). CD2-targeting immunotherapy is a promising treatment for organ transplant recipients and patients suffering from autoimmune diseases [Reviewed in (1)]. Siplizumab is a monoclonal anti-CD2 IgG1 antibody (anti-CD2 IgG1 mAb) that is currently undergoing clinical trials in the field of transplantation (ClinicalTrials.gov Identifier: NCT04311632). While the potent *in vitro* immune modulatory effects of siplizumab on T cells have been documented (2, 3), no studies published to date address the *in vitro* effects of siplizumab on NK cells. Previous *in vivo* evidence showed siplizumab-induced peripheral T and NK cell depletion in primates (4) and human patients (5). Evidence suggests that antibody-dependent cytotoxicity is one depletory mechanism induced by siplizumab; however, the direct effects of siplizumab on NK cells remain incompletely characterized (3, 6).

NK cells express activating and inhibitory receptors. The balance of signaling through both sets of receptors is integrated and determines whether mature NK cells remain in a resting state or become activated (7). Thus, NK cell activation can be elicited by a reduction of inhibitory signaling, an increase in activation signaling, or a combination of both.

Among activating NK cell receptors, CD16 is one whose signaling can induce NK cell activation in isolation. Most remaining activating NK cell receptors require activation in conjunction to elicit NK cell activation. CD16a is a low affinity Fc γ receptor (FcγR) and is also known as FcγRIIIA. NK cell binding to target-bound IgG antibodies *via* CD16a promotes antibody-dependent cell-mediated cytotoxicity (ADCC). Other activating NK cell receptors include NKG2D, certain killer cell immunoglobulin-like receptors (KIRs) and NKp46 which bind to tumor antigens, foreign HLA and viral proteins, respectively. Most prominent among inhibitory NK cell receptors are KIRs which recognize self-HLA (8). Antibody-independent target cell killing by NK cells is commonly termed natural or spontaneous NK cell cytotoxicity. In addition to FcγRIIIA, certain NK cell subsets can express two forms of CD32/FcγRII, FcγRIIB, and FcγRIIC (9).

A critical event preceding NK cell cytotoxicity is stable target cell conjugation *via* cell-cell adhesion molecules (10). Once stable target cell conjugation has occurred and the NK cell immunological synapse (NKIS) has formed, actin cytoskeleton rearrangement proceeds to facilitate transport of lytic vesicles to the NKIS and subsequent release to achieve target cell killing. Examples of adhesion molecules involved in NK-target cell conjugation and NKIS formation are LFA-1 (8) and CD2 (10) binding to ICAM-1 and LFA3 on the target cell, respectively. Even in natural NK cell cytotoxicity CD16 is enriched in the NKIS through interaction with CD2. Abrogation of this interaction between CD2 and CD16 markedly decreases natural NK cell cytotoxicity but does not affect NK-mediated ADCC (11). CD2 has two Ig domains, one membrane distal domain and one membrane proximal domain. The membrane distal domain binds LFA3 but to date the site on CD2 which interacts with CD16 has not been reported. Depending on where CD16 binds to CD2, simultaneous binding of CD2 to both CD16 and LFA3 may be conceivable. The role of CD2 in NK cell biology is incompletely characterized.

Similar to what has been observed during T cell-antigen presenting cell (APC) conjugation, CD2 accumulates in the immunological synapse that NK cells form upon target cell conjugation. While the CD2-CD58 interaction has traditionally been known for its role in cellular adhesion, recent evidence has demonstrated that it also plays an important role in recruiting and organizing activating receptors to the immunological synapse (11, 12).

This study aimed to characterize the effects of siplizumab on NK cell activation in mixed lymphocyte reaction (MLR) and in pure NK cell culture. Further, potential effects of CD2 blockade on natural cytotoxicity and ADCC were investigated.

## Materials and Methods

### Generation of Fc-Silent Anti-CD2 Antibodies

Siplizumab (humanized anti-CD2 IgG1κ) is an investigational drug and was provided by the manufacturer (ITB-Med, Stockholm, Sweden).

Deglycosylated (DG) siplizumab was produced using GlycINATOR^®^ (EndoS2; Genovis #A0-GL1-020) according to the manufacturer’s instructions (Genovis AB, Lund, Sweden).

Fc-silent (FcS) anti-CD2 mAbs were generated *via* introduction of amino acid substitutions into the sequence of siplizumab or standard IgG2/IgG4 frameworks. All Fc-silent antibodies had identical light chains and complementarity-determining regions and were produced by Wuxi Biologics Limited (Wuxi, China). Genetic sequences for each antibody were cloned into plasmids followed by transient transfection and production in Chinese hamster ovary (CHO-K1) cells. Anti-CD2 mAbs were purified using protein A and IEX/SEC columns to at least 95% purity and endotoxin levels below 1 EU/mg. Fc-silent siplizumab (FcS anti-CD2 IgG1) was generated by introducing the commonly known LALA-PG mutations to equivalent sites in siplizumab (13, 14). FcS anti-CD2 IgG2 was generated by introducing the following mutations at equivalent sites in a standard IgG2 framework: V234A, G237A P238S, H268A, V309L, A330S, P331S (15). FcS anti-CD2 IgG4 was generated by introducing the following mutations at equivalent sites in a standard IgG4 framework: S228P, L235E, P329G (16, 17).

### Surface Plasmon Resonance CD2 Binding

Interaction experiments were performed using a Biacore T200 instrument (Cytiva Life Sciences/Biacore, Uppsala, Sweden). IgG was immobilized to a surface density of 200-1300 RU using standard amine coupling and CM5 biosensor chips (Cytiva Life Sciences/Biacore, Uppsala, Sweden) with a 10 mM HEPES buffer at pH 7.4, with 150 mM NaCl, 0.5 mM EDTA, 0.05% Tween20 running buffer. Each antibody was immobilized at least twice.

All interaction experiments were performed at 25°C. Recombinant truncated human CD2 (Sino Biological, Beijing, China) was diluted in two-fold dilution series with the highest concentration being 5 nM and injected for 180 s over immobilized IgG. After 15 min of dissociation, the sensor chip was regenerated by 30 s injection of 10 mM glycine pH 1.7. Sensorgrams from reference surfaces and blank injections were subtracted from the raw data prior to data analysis. A two-state (induced fit) interaction model was fitted to the data using T200 evaluation software v3.0 (Cytiva Life Sciences/Biacore, Uppsala, Sweden) to determine kinetic rate constants and affinities.

### SPR FcγR Binding

Recombinant truncated human CD2 (Sino Biological, Beijing, China) was covalently immobilized by amine coupling to CM5 biosensor chips (Cytiva Life Sciences/Biacore, Uppsala, Sweden). IgG was captured to amine coupled CD2. All experiments were performed using a Biacore T200™ instrument (Cytiva Life Sciences/Biacore, Uppsala, Sweden) at 25°C. The characterization of compound interactions were conducted in a 10 mM HEPES

buffer at pH 7.4, with 150 mM NaCl, 0.5 mM EDTA, 0.05% Tween20. Fcγ receptors (R&D systems, Minneapolis USA) were injected for 120 s in multi-cycle or single-cycle experiments in concentration series up to 0.5 μM over CD2-captured IgGs. The R131 isoform of FcγRIIA and V176 isoform of FcγRIIIA were used for all experiments. CD2 surfaces were regenerated by a 120 s injection of 50 mM NaOH. Sensorgrams were double-referenced (reference surface, blanks) prior to global analysis using a 1:1 interaction model or a heterogeneous binding model. For weaker interactions with fast dissociation the affinity was determined by steady state analysis using a sum of a Langmuir isotherm and a linear term (compensating for non-specific interaction). Kinetic parameters were determined as average values based on 2–3 replicate experimental series.

### Cell-Based FcγR Signaling Assay

Jurkat reporter cell lines (Promega Corp., Madison, Wisconsin USA) transfected with FcγRI, FcγRIIA or FcγRIIIA, respectively, were incubated with serial dilutions of each antibody agent for 23 h. Binding of a target-bound antibody through the respective FcγR induced expression of luciferase in reporter cells. Luciferase expression was detected after 23 h of incubation at 37°C 5% CO_2_ *via* addition of luciferase assay substrate (Promega Corp., Madison, Wisconsin USA) and luminescence measurement using a Synergy HTX multiplate reader (BioTek, Winooski, Vermont USA).

### Isolation of Peripheral Blood Mononuclear Cells

Peripheral blood mononuclear cells (PBMC) were isolated *via* Ficoll^®^ Paque Plus (Cytiva Life Sciences, Uppsala, Sweden) density gradient centrifugation from buffy coats. Buffy coats were obtained from healthy donors *via* Uppsala University Hospital blood bank (Uppsala, Sweden) as well as Karolinska University Hospital blood bank (Stockholm, Sweden) and PBMC isolation was carried out within 24 h of blood collection.

### CD2 Expression Analysis

Resting PBMC were stained and analyzed for target antigen expression. Subsequently, PBMC in autologous culture were activated *via* CD28/CD3 beads (Miltenyi, Bergisch Gladbach, Germany; bead to cell ratio 5:1) for 48 h and cultured in 10% heat-inactivated fetal bovine serum (FBS; Gibco, Thermo Fisher Scientific Inc., Waltham, USA) in RPMI-1640 ATCC Mod. supplemented with 50 U/ml Streptomycin and Penicillin (Gibco, Thermo Fisher Scientific Inc., Waltham, USA) and target antigen expression was investigated.

PBMC were washed twice in saline solution. Samples were incubated with Fc-receptor binding inhibitor (Invitrogen; Thermo Fisher Scientific Inc., Waltham, USA) and then washed twice in FBS stain buffer (BD BioSciences, San Diego, USA) followed by incubation with 10 µg/ml siplizumab for 20 min. Unbound antibody was removed before staining with secondary anti-human IgG Fc BV421 (BioLegend, San Diego, USA; Clone HP6017). Unbound secondary antibody was removed before surface staining for T cell subpopulations, regulatory T cells or B and NK cells. All antibodies listed below were purchased from BD Biosciences (San Diego, USA) if not indicated otherwise.

Samples to be used for analysis of T cell subpopulation distribution were stained with anti-CD3 PerCP (Clone SK7), anti-CD4 FITC (Clone SK3) and anti-CD8 PE (Clone SK1), with anti-CCR7 Alexa647 (Clone 3D12) and anti-CD45RA APC-H7 (Clone HI100) (Naïve T cells: CCR7^+^ CD45RA^+^; Central memory T cells: CCR7^+^ CD45RA^-^; Effector memory T cells: CCR7^-^ CD45RA^-^; Terminally-differentiated effector memory T cells (Temra): CCR7- CD45RA^+^).

Samples that analyzed Tregs were stained anti-CD4 FITC (Clone RPA-T4), anti-CD25 PE (Clone M-A251), anti-CD45RA APC-H7 (Clone HI100), anti-CD127 PerCP-Cy5.5 (Clone HIL-7R-M21) and anti-FoxP3 Alexa647 (Clone 259D/C7). They were permeabilized using BD Human FoxP3 Buffer Set (BD BioSciences, San Diego, USA) according to the vendor’s protocol. Tregs were identified as CD4^+^ CD127^-^ CD25^+^ FoxP3^+^.

NK/B cell panel consisted of anti-CD16 FITC (Clone 3G8), anti-CD56 PE (Miltenyi, Bergisch Gladbach, Germany; Clone REA196), anti-CD3 APC (Clone SK7) anti-CD14 APC-H7 (Clone M5E2), and anti-CD20 PerCP (Clone SJ25C1).

Samples were stained in the dark at 4°C and were acquired using a FACSVerse flow cytometer (BD Biosciences, San Diego, USA). Post-acquisition editing and data analysis was conducted using FlowJo^®^ 10.5.3 software (FlowJo LLC, Ashland, USA).

### FcγR Expression Analysis

NK cells were isolated *via* negative magnetic bead selection using NK cell isolation kit (Miltenyi, Bergisch Gladbach, Germany) according to the manufacturer’s instructions. A fraction of NK cells were washed twice in saline solution and stained with anti-CD56 BV421 (Clone NACM16.2), anti-CD3 VioGreen (Miltenyi, Bergisch Gladbach, Germany; Clone REA613), anti-CD16 FITC (Miltenyi, Bergisch Gladbach, Germany; Clone REA423), anti-CD32 PE (Miltenyi, Bergisch Gladbach, Germany; Clone REA997), and anti-CD64 APC (Miltenyi, Bergisch Gladbach, Germany; Clone REA978). Samples were stained in the dark at 4°C and were acquired using a FACSCelesta flow cytometer (BD Biosciences, San Diego, USA). Post-acquisition editing and data analysis was conducted using FlowJo^®^ 10.6.2 software (FlowJo LLC, Ashland, USA). NK cells were defined as CD3^-^ CD56^+^ and/or CD16^+^ lymphocytes.

### Autologous Lymphocyte Culture and Mixed Lymphocyte Reaction

For analysis of NK cell activation over time in the presence of siplizumab and DG siplizumab, equal amounts of PBMC from each donor were stained with violet proliferation dye 450 (BD Biosciences, San Diego, USA; VPD450) for another experiment. Cells were resuspended in 10% heat-inactivated FBS (Gibco, Thermo Fisher Scientific Inc., Waltham, USA) in RPMI-1640 ATCC Mod. supplemented with 50 U/ml Streptomycin and Penicillin (Gibco, Thermo Fisher Scientific Inc., Waltham, USA), respectively. Resuspended PBMC were dispensed into round-bottom 96-well cell culture plates and pure medium (no antibody controls) or one of the respective antibody agents diluted in cell culture medium (10 µg/ml) was added to a final concentration of 2x10^6^ cells per ml (final volume 200 µl). ALCs and MLRs were then incubated at 37°C 5% CO_2_ for 1, 2, 4, and 7 days, respectively. On each day, cells were washed twice in saline solution and incubated with Fc-receptor binding inhibitor (Invitrogen; Thermo Fisher Scientific Inc., Waltham, USA) followed by staining with anti-CD16 FITC (Clone 3G8), anti-CD56 PE (Miltenyi, Bergisch Gladbach, Germany; Clone REA196), anti-CD3 PerCP-Cy5.5 (Clone SP34-2), anti-CD69 APC (Clone FN50) and anti-CD14 APC-H7 (Clone MφP-9). NK cells were identified as CD3^-^ CD14^-^ CD56^+^ and/or CD16^+^ lymphocytes. All flow cytometry antibodies were purchased from BD Biosciences (San Diego, USA) if not indicated otherwise. Samples were stained in the dark at 4°C and were acquired using a FACSVerse flow cytometer (BD Biosciences, San Diego, USA). Post-acquisition editing and data analysis was conducted using FlowJo^®^ 10.5.3 software (FlowJo LLC, Ashland, USA).

For analysis of NK cell activation after 7 days in the presence of different anti-CD2 variants, equal amounts of PBMC from each donor were stained with VPD450 for another experiment. Cells were resuspended in 10% heat-inactivated FBS (Gibco, Thermo Fisher Scientific Inc., Waltham, USA) in AIM-V medium supplemented with 50 µg/ml streptomycin sulfate and 10 µg/ml gentamicin sulfate (Gibco, Thermo Fisher Scientific Inc., Waltham), respectively. Resuspended PBMC were dispensed into round-bottom 96-well cell culture plates and pure medium (No antibody controls) or one of the respective antibody agents diluted in cell culture medium (10–0.0001 µg/ml) was added to a final concentration of 2x10^6^ cells per ml (final volume 200 µl). MLRs were then incubated at 37°C 5% CO_2_ for 7 days, respectively. Fresh culture medium (100 µl) was added to each well on day 5. On day 7, cells were washed twice in saline solution and incubated with Fc-receptor binding inhibitor (Invitrogen; Thermo Fisher Scientific Inc., Waltham, USA) followed by staining with anti-CD3 VioGreen (Miltenyi, Bergisch Gladbach, Germany; Clone REA613), anti-CD45RA BV650 (Clone HI100), anti-CD69 BV786 (Clone FN50), anti-CD8 BB550 (Clone RPA-T8), anti-CD56 PE (Miltenyi, Bergisch Gladbach, Germany; Clone REA196), anti-CD2 PE-CF594 (Clone RPA-2.10; Note: This clone binds a different CD2 epitope than siplizumab), anti-CCR7 APC (BioLegend, San Diego, USA; Clone G043H7), and anti-CD4 APC-Vio770 (Miltenyi, Bergisch Gladbach, Germany; Clone REA623). NK cells were identified as CD3^-^ CD56^+^ lymphocytes. Remaining colors were used for another experiment. All flow cytometry antibodies were purchased from BD Biosciences (San Diego, USA) if not indicated otherwise. Samples were stained in the dark at 4°C and were acquired using a FACSCelesta flow cytometer (BD Biosciences, San Diego, USA). Post-acquisition editing and data analysis was conducted using FlowJo^®^ 10.6.2 software (FlowJo LLC, Ashland, USA).

### NK Fratricide

NK cells were isolated from PBMC *via* negative MACS selection using NK cell isolation kit (Miltenyi, Bergisch Gladbach, Germany). NK cells were resuspended in 10% heat-inactivated FBS (Gibco, Thermo Fisher Scientific Inc., Waltham, USA) in AIM-V medium supplemented with 50 µg/ml streptomycin sulfate and 10 µg/ml gentamicin sulfate (Gibco, Thermo Fisher Scientific Inc., Waltham) as well as 500 IU/ml human IL-2 (Miltenyi, Bergisch Gladbach, Germany) and, depending on yield, dispensed into round-bottom 96-well cell culture plates at a final density between 1.25x10^6^ and 2.0x10^6^ NK cells per ml. Further, pure medium (No antibody controls) or one of the respective antibody agents diluted in cell culture medium (final concentration 10–0.001 µg/ml) was added followed by overnight incubation at 37°C 5% CO_2_. Subsequently, NK cells were washed in saline solution followed by incubation with Fc-receptor binding inhibitor (Invitrogen; Thermo Fisher Scientific Inc., Waltham, USA) and staining with anti-CD3 VioGreen (Miltenyi, Bergisch Gladbach, Germany; Clone REA613), anti-CD107a BV786 (BioLegend, San Diego, USA; Clone H4A3), anti-CD16 FITC (Miltenyi, Bergisch Gladbach, Germany; Clone REA423), anti-CD56 PE (Miltenyi, Bergisch Gladbach, Germany; Clone REA196), 7-AAD (Invitrogen; Thermo Fisher Scientific Inc., Waltham, USA) and anti-CD69 APC (Miltenyi, Bergisch Gladbach, Germany; Clone REA824). NK cells were identified as CD3^-^ CD56^+^ and/or CD16^+^ lymphocytes. Samples were stained in the dark at 4°C and were acquired using a FACSCelesta flow cytometer (BD Biosciences, San Diego, USA). Post-acquisition editing and data analysis was conducted using FlowJo^®^ 10.6.2 software (FlowJo LLC, Ashland, USA).

### Natural Cytotoxicity Assay

NK cells were isolated from PBMC *via* negative MACS selection using NK cell isolation kit (Miltenyi, Bergisch Gladbach, Germany).

For runs with short pre-incubation, NK cells were resuspended in 10% heat-inactivated FBS (Gibco, Thermo Fisher Scientific Inc., Waltham, USA) in AIM-V medium supplemented with 50 µg/ml streptomycin sulfate and 10 µg/ml gentamicin sulfate (Gibco, Thermo Fisher Scientific Inc., Waltham) and dispensed into round-bottom 96-well cell culture plates followed by resting at 37°C 5% CO_2_ overnight. Following, pure medium (no antibody controls) or one of the respective antibody agents diluted in cell culture medium (final concentration 10–0.001 µg/ml) were added followed by 30 min incubation at 37°C 5% CO_2_ before addition of HLA class I-negative target cells (SPI-801, DSMZ Cat. ACC 86; NK cell to target cell ratio between 1:1 and 1:2 depending on yield of MACS isolation). Addition of target cells was followed by incubation at 37°C 5% CO_2_ for 2 h and subsequent flow cytometry staining as described in *NK Fratricide* section.

For runs with pre-incubation of NK cells with antibody, NK cells were resuspended in in 10% heat-inactivated FBS (Gibco, Thermo Fisher Scientific Inc., Waltham, USA) in AIM-V medium supplemented with 50 µg/ml streptomycin sulfate and 10 µg/ml gentamicin sulfate (Gibco, Thermo Fisher Scientific Inc., Waltham) as well as 500 IU/ml human IL-2 (Miltenyi, Bergisch Gladbach, Germany). Further, pure medium (No antibody controls) or one of the respective antibody agents diluted in cell culture medium (final concentration 10–0.001 µg/ml) were added followed by incubation at 37°C 5% CO_2_ for 2 days. On day 2, HLA class I-negative target cells were added (SPI-801; NK cell to target cell ratio between 1:1 and 1:2 depending on yield of MACS isolation) followed by incubation at 37°C 5% CO_2_ for 2 h and subsequent flow cytometry staining as described in *NK Fratricide* section.

### Rituximab-Induced ADCC Assay

NK cells were isolated from PBMC *via* negative MACS selection using NK cell isolation kit (Miltenyi, Bergisch Gladbach, Germany). resuspended in 10% heat-inactivated FBS (Gibco, Thermo Fisher Scientific Inc., Waltham, USA) in AIM-V medium supplemented with 50 µg/ml streptomycin sulfate and 10 µg/ml gentamicin sulfate (Gibco, Thermo Fisher Scientific Inc., Waltham) and dispensed into round-bottom 96-well cell culture plates. Further, pure medium (No antibody controls) or one of the respective anti-CD2 antibody agents diluted in cell culture medium (final concentration 10 µg/ml) were added followed by overnight incubation at 37°C 5% CO_2_. The next day Daudi target cells (DSMZ Cat. ACC 78; CD20^+^; NK to Daudi ratio 4:1) and Rituximab (final concentration 0.0001–1 µg/ml) were added followed by 2 h of incubation at 37°C 5% CO_2_. Subsequently, cells were washed in saline solution and incubated with Fc-receptor binding inhibitor (Invitrogen; Thermo Fisher Scientific Inc., Waltham, USA) followed by staining with anti-CD56 BV421 (BD Biosciences, San Diego, USA; Clone NCAM16.2), anti-CD3 VioGreen (Miltenyi, Bergisch Gladbach, Germany; Clone REA613), anti-CD107a BV786 (BioLegend, San Diego, USA; Clone H4A3), anti-CD16 FITC (Miltenyi, Bergisch Gladbach, Germany; Clone REA423), anti-CD2 PE-CF594 (BD Biosciences, San Diego, USA; Clone RPA-2.10), anti-CD69 APC (Miltenyi, Bergisch Gladbach, Germany; Clone REA824), and anti-CD19 APC-H7 (BD Biosciences, San Diego, USA; Clone SJ25C1). NK cells were identified as CD3^-^ CD56^+^ and/or CD16^+^ lymphocytes. Samples were stained in the dark at 4°C and were acquired using a FACSCelesta flow cytometer (BD Biosciences, San Diego, USA). Post-acquisition editing and data analysis was conducted using FlowJo^®^ 10.6.2 software (FlowJo LLC, Ashland, USA).

### Graphs and Statistical Analysis

Visualization of results and statistical analysis of underlying data were carried out using GraphPad Prism 8 software (GraphPad Software, San Diego). Data were analyzed using repeated-measure one-way ANOVA followed by Dunnett’s multiple comparison test or two-way ANOVA followed by Dunnett’s multiple comparison test unless specified otherwise. Untreated controls served as the comparator if not specified otherwise.

## Results

### CD2 and Fc Gamma Receptor Binding

Anti-CD2 antibody binding to CD2 and FcγR binding was characterized *via* surface plasmon resonance (SPR). As shown in **Table 1**, all tested antibodies displayed a similar CD2 binding affinity with an affinity constant (K_D_) of circa 0.9–1.5 nM. Siplizumab binds FcγRI, FcγRIIA and FcγRIIIA. In contrast, deglycosylated (DG) siplizumab binds FcγRI but not FcγRIIA and FcγRIIIA. All three Fc-silent anti-CD2 antibodies did not display detectable FcγR affinity.

**Table 1:** Affinity of different anti-CD2 antibodies for CD2 and Fc gamma receptor (FcγR) I, IIA and IIIA.

*K_D_* ^(a)^	Siplizumab	DG^(b)^ siplizumab	FcS^(c)^ IgG1	FcS IgG2	FcS IgG4
CD2	1.2	1.2	1.5	0.9	1.4
Fc gamma RI	0.2	1.13	>2000	>2000	>2000
Fc gamma RIIA	579	>5000	>5000	>5000	>5000
Fc gamma RIIIA	131	>2000	>5000	>5000	>5000

K_D_ of siplizumab, DG siplizumab, FcS anti-CD2 IgG1, FcS anti-CD2 IgG2, and FcS anti-CD2 IgG4 for CD2, FcγRI, FcγRIIA, and FcγRIIIA was measured via surface plasmon resonance.

^(a)^K_D_, Affinity constant; ^(b)^DG, deglycosylated; ^(c)^FcS, Fc-silent.

**Figure 1:**
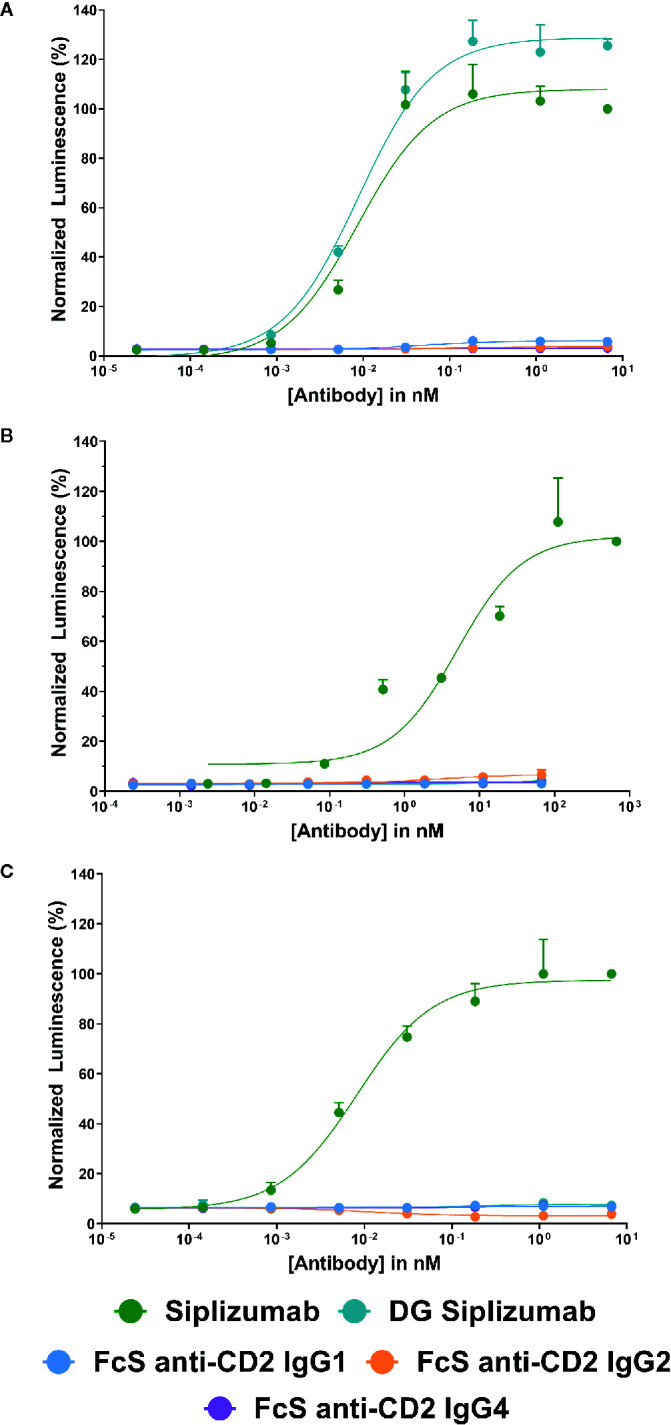
Jurkat reporter cells (Promega) stably expressing Fc γ receptor (FcγR) I, FcγRIIA or FcγRIIIA, respectively, were incubated with increasing concentrations of anti-CD2 antibody. Data normalized to luminescence induced by the highest concentration of siplizumab (mean ± SD; n=3). Reporter cells bind the Fc-fragment of a target-bound IgG antibody with their FcγR which induces expression of a luciferase reporter gene resulting in a luminescence signal upon addition of assay substrate (Promega). Cells were incubated with increasing doses of siplizumab, deglycosylated (DG) siplizumab, Fc-silent (FcS) anti-CD2 IgG1, FcS anti-CD2 IgG2 and FcS anti-CD2 IgG4, respectively. **(A)** Cell-based FcγRI signaling assay. **(B)** Cell-based FcγRIIA signaling assay. **(C)** Cell-based FcγRIIIA signaling assay.

To confirm that FcγR-binding results in FcγR-induced signaling, each antibody was tested in a cell-based FcγR signaling assay (**Supplementary Tables S1–S3**). As shown in **Figure 1**, consistent with SPR results siplizumab induced signaling through FcγRI, FcγRIIA and FcγRIIIA. In contrast, DG siplizumab only induced signaling through FcγRI. None of the Fc-silenced anti-CD2 variants induced FcγR-mediated signaling.

### CD2 and FcγR Expression on NK Cells

**Figure 2:**
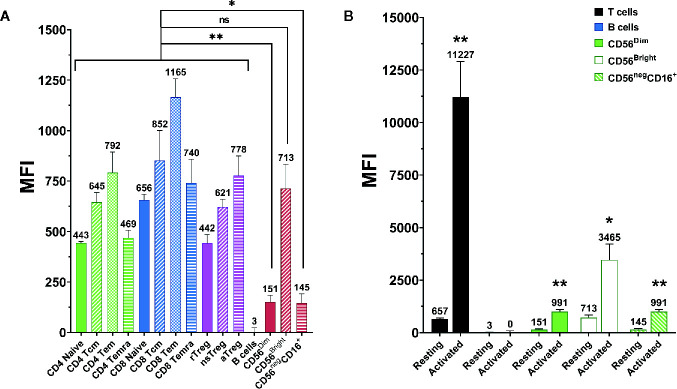
CD2 expression on different lymphocyte (sub-)populations. Data displayed as average median fluorescence intensity ± SEM (n = 5; *p < 0.05, **p < 0.01). **(A)** CD2 expression on different T and NK cell subpopulations. CD56^bright^ NK cells express CD2 at levels comparable to that of T cells. In contrast, CD56^dim^, and CD56^neg^ NK cells express significantly lower levels of CD2 (p=0.0074 and p=0.0111, respectively, repeated-measure one-way ANOVA followed by Dunnett’s multiple comparison test). **(B)** CD2 expression on resting and activated lymphocyte populations. CD2 expression was markedly upregulated after activation on T cells (p=0.0034), CD56^dim^ NK cells (p=0.0024), CD56^bright^ NK cells (p=0.017), and CD56^neg^ CD16^+^ NK cells (0.0029). No difference was observed on B Cells (two-tailed paired t-test).

CD2 expression on NK cells was measured *via* flow cytometry. As shown in **Figure 2A**, NK cells tend to have a lower CD2 expression than T cells. While CD56^bright^ NK cells express CD2 at comparable levels as the average T cell, CD56^dim^ and CD56^neg^ CD16^+^ NK cells express CD2 at significantly lower levels (p=0.0074 and p=0.0111, respectively; Repeated-measure one-way ANOVA followed by Dunnett’s multiple comparison test; **Supplementary Table S4**). Nevertheless, NK cells show notable CD2 expression when compared to CD2^-^ cell types, e.g. B cells.

Additionally, CD2 expression on T and NK cells was measured before and after activation of PBMC with anti-CD3 and anti-CD28 antibody-conjugated microbeads (**Supplementary Table S5**). As displayed in **Figure 2B**, CD2 expression is significantly elevated after activation on T cells (p=0.0034), CD56^dim^ NK cells (p=0.0024), CD56^bright^ NK cells (p=0.017) and CD56^-^ CD16^+^ NK cells (p=0.0029; two-tailed paired t-test).

**Figure 3:**
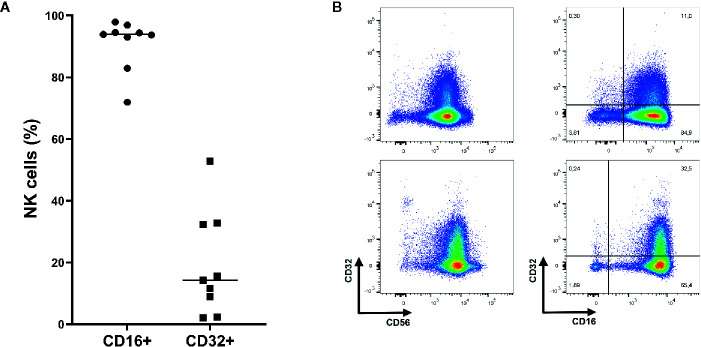
Expression of Fc gamma receptor (FcγR) II (CD32) and FcγRIII (CD16) on NK cells. **(A)** On average 91.0% of NK cells express FcγRIII while 19.2% express FcγRII (n=9). Mean shown as horizontal line. **(B)** Representative plots of FcγRII and FcγRIII expression on NK cells (CD3^-^ CD56^+^ and/or CD16^+^ lymphocytes). Upper: Representative plots of NK cells from donor with relatively low CD32 expression. Lower: Representative plots of NK cells from donor with relatively high CD32 expression.

As shown in **Figure 3**, almost all NK cells (91.0%) expressed FcγRIIIA (CD16) while FcγRII expression was heterogeneous (19.2%, CD32). NK cells did not express FcγRI (data not shown).

### Effect of CD2 Blockade on Antibody-Mediated and Natural NK Cell Activation in Autologous and Mixed Lymphocyte Culture

**Figure 4:**
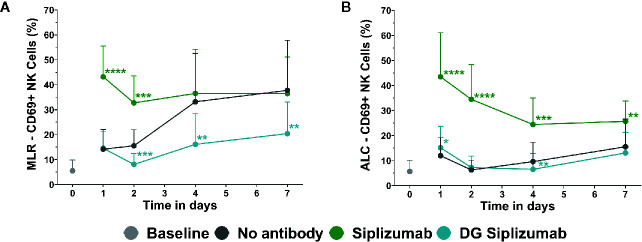
CD69 Expression on NK cells in mixed lymphocyte reaction (MLR) and autologous lymphocyte culture (ALC) over time. CD69 Expression without addition of antibody (No antibody) and with added antibody was assessed at baseline (day 0) and after one, two, four and 7 days *via* flow cytometry. Significance testing was conducted *via* two-way ANOVA with untreated controls (no antibody) serving as the comparison data set (*p < 0.05, **p < 0.01, ***p < 0.001, ****p < 0.0001). Siplizumab and deglycosylated (DG)**** siplizumab were used at 10 µg/ml, a dose which has previously determined to saturate target antigen over 7 days. Data displayed as mean ± SD (n=12). **(A)** Percentage of CD69^+^ NK cells in MLR over time. **(B)** Percentage of CD69^+^ NK cells in ALC over time.

To investigate the effect of siplizumab on NK cell activation, autologous lymphocyte culture (ALC) and MLR were performed with a saturating dose of siplizumab. As shown in **Figure 4**, siplizumab significantly increased the percentage of CD69+ NK cells (% CD69^+^ NK cells) after 1 (p<0.0001) and 2 (p=0.0001) days of MLR as well as after 1 (p<0.0001), 2 (p<0.0001), 4 (p=0.0006), and 7 days (p=0.0082) of ALC (**Supplementary Tables S6 **and** S7**). On days 4 and 7 of MLR an increase in % CD69^+^ NK cells in untreated controls (no antibody) was observed. This likely derived from NK cells reacting against allogeneic HLA and/or absence of self-HLA on PBMC from the other donor. As shown in **Figure 4**, DG siplizumab significantly reduced % CD69+ NK cells after 4 (p=0.0012) and 7 (p=0.0035) days (Repeated-measure two-way ANOVA followed by Dunnett’s multiple comparison test). Addition of DG siplizumab did not meaningfully change the percentage of CD69^+^ NK cells in ALC.

**Figure 5:**
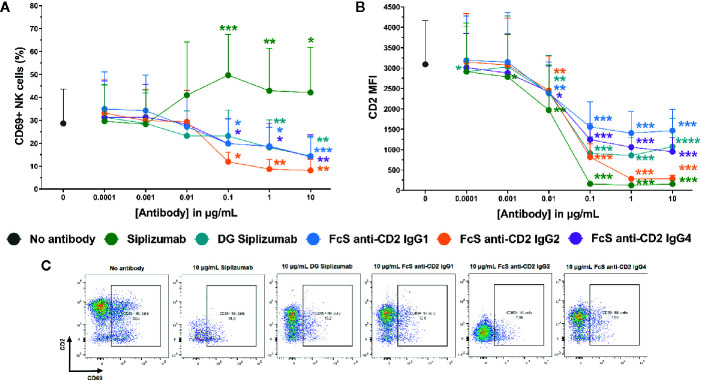
CD69 and CD2 expression on NK cells after 7 days of mixed lymphocyte reaction. Significance testing was conducted *via* one-way ANOVA with untreated controls (no antibody) serving as the comparison data set (*p < 0.05, **p < 0.01, ***p < 0.001, ****p < 0.0001). Siplizumab, deglycosylated (DG) siplizumab, Fc-silent (FcS) anti-CD2 IgG1, FcS anti-CD2 IgG2 and FcS anti-cD2 IgG4 were used at 0.0001–10 µg/ml. Data displayed as mean percentage of CD69^+^ NK cells ± SD **(A)** or average median CD2 fluorescence intensity +SD [CD2 MFI, **(B)**; n=9). **(C)** Representative dot plots of CD2 and CD69 expression on NK cells in untreated controls (no antibody) or 10 µg/ml anti-CD2.

To further characterize the inhibition of NK cell activation in MLR by CD2 blockade, dose titrations from 0.0001 to 10 µg/ml were performed and CD69 expression on NK cells was analyzed after 7 days of MLR. MLRs were incubated with siplizumab, DG siplizumab, FcS anti-CD2 IgG1, FcS anti-CD2 IgG2, or FcS anti-CD2 IgG4. As displayed in **Figure 5A**, doses of siplizumab induced a significant increase in % CD69^+^ NK cells at 0.1 µg/ml (p=0.0003), 1 µg/ml (p=0.0018), and 10 µg/ml (p=0.0256) relative to untreated controls (No antibody). In contrast, DG siplizumab induced a significant decrease of % CD69^+^ NK cells at 1 µg/ml (p=0.0012) and 10 µg/ml (p=0.006). FcS anti-CD2 IgG1, FcS anti-CD2 IgG2, and FcS anti-CD2 IgG4 significantly reduced % CD69^+^ NK cells at 0.1 µg/ml (p=0.0178, p=0.0122, p=0.0234, respectively), 1 µg/ml (p=0.0311, p=0.0039, p=0.0388, respectively), and 10 µg/ml (p=0.0008, p=0.0027, p=0.0023, respectively) (Repeated-measure one-way ANOVA followed by Dunnett’s multiple comparison test; **Supplementary Table S8**).

Additionally, median CD2 fluorescence intensity (CD2 MFI) on NK cells was measured to assess if siplizumab or FcS anti-CD2 mAbs affect CD2 expression (**Figures 2B, C**). Siplizumab induced a significant decrease of CD2 MFI on NK cells at 0.001–10 µg/ml (p ≤ 0.0354) relative to untreated controls. Varying degrees of significant CD2 downregulation were observed with DG siplizumab (p ≤ 0.0064), FcS anti-CD2 IgG1 (p ≤ 0.0050), FcS anti-CD2 IgG2 (p ≤ 0.0091), and FcS anti-CD2 IgG4 (p ≤ 0.0142) at 0.01–10 µg/ml (Repeated-measure one-way ANOVA followed by Dunnett’s multiple comparison test; **Supplementary Table S9**).

### NK Fratricide

**Figure 6:**
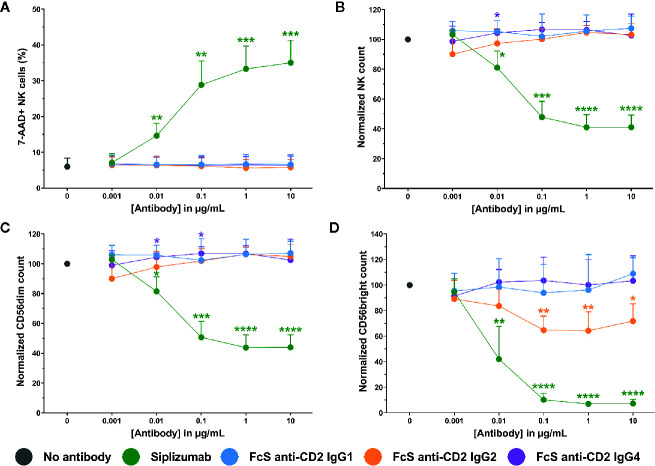
NK cell fratricide. Purified NK cells were incubated with no antibody, 0.001–10 µg/ml Siplizumab or 0.001–10 µg/ml Fc-silent (FcS) anti-CD2 IgG antibodies overnight. Significance testing was conducted *via* one-way ANOVA with untreated controls (no antibody) serving as the comparison data set (N=6; *p < 0.05, **p < 0.01, ***p < 0.001, ****p < 0.0001). **(A)** NK cell lysis. Mean percentage of 7-AAD+ NK cells +SD. **(B)** NK cell count. Mean normalized NK cell count +SD. **(C)** CD56^dim^ NK cell count. Mean normalized CD56^dim^ NK cell count +SD. **(D)** CD56^bright^ NK cell count. Mean normalized CD56^bright^ NK cell count +SD.

To test whether siplizumab can induce NK cell fratricide, purified NK cells were cultured in the presence of increasing doses of siplizumab or Fc-silent anti-CD2 mAbs. As shown in **Figure 6**, siplizumab significantly increased NK cell lysis in purified NK cell culture at 0.01–10 µg/ml (p ≤ 0.0018) relative to untreated controls. Furthermore, siplizumab induced a significant dose-dependent depletion of total NK cells (p ≤ 0.0301), CD56dim NK cells (p ≤ 0.0191) and CD56bright NK cells (p ≤ 0.0093) at 0.01–10 µg/ml when compared to untreated controls. In contrast, FcS anti-CD2 mAbs did not induce dose-dependent NK cell lysis or depletion in pure NK culture, except FcS anti-CD2 IgG2 which induced a mild but statistically significant depletion of CD56bright NK cells at 0.1–10 µg/ml (p ≤ 0.0132; Repeated-measure one-way ANOVA followed by Dunnett’s multiple comparison test; **Supplementary Tables S10–S13**).

**Figure 7:**
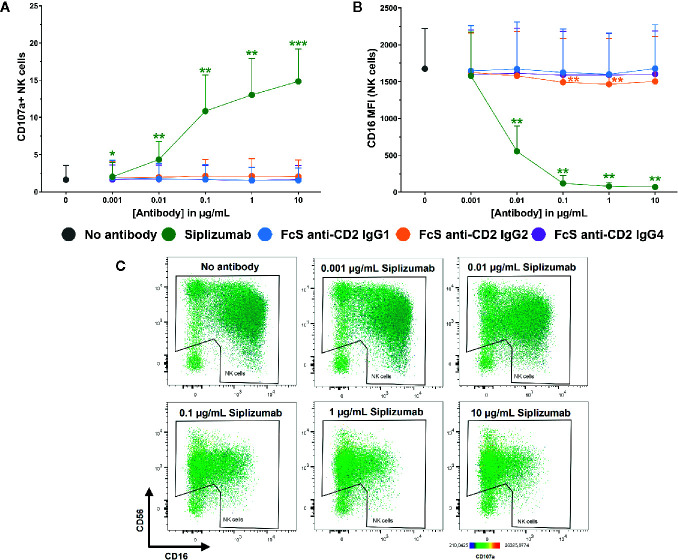
NK cell degranulation and ADCC in purified NK cell culture. Purified NK cells were incubated without antibody, with 0.001–10 µg/ml Siplizumab or 0.001–10 µg/ml Fc-silent (FcS) anti-CD2 IgG antibodies. Significance testing was conducted *via* one-way ANOVA with untreated controls (no antibody) serving as the comparison data set (N=6; *p < 0.05, **p < 0.01, ***p < 0.001). **(A)** NK cell degranulation. Mean percentage of CD107a^+^ NK cells +SD. **(B)** CD16 expression on NK cells. Average median CD16 fluorescence intensity +SD on NK cells. **(C)** Representative heatmap dot plots illustrating CD16 downregulation and NK cell degranulation with increasing doses of Siplizumab in purified NK cell culture.

Additionally, **Figure 7** shows that siplizumab induced a significant dose-dependent increase in NK cell degranulation (p ≤ 0.0114; CD107a^+^ NK cells) and a strong decrease of CD16 expression on NK cells (p ≤ 0.0022), indicative of ADCC. No FcS anti-CD2 mAb induced degranulation and only FcS anti-CD2 IgG2 induced a minor but statistically significant decrease in CD16 MFI at 0.1–1 µg/ml (p ≤ 0.0269; Repeated-measure one-way ANOVA followed by Dunnett’s multiple comparison test; **Supplementary Tables S14** and** S15**).

### Natural NK Cytotoxicity

To test the effect of CD2 blockade on natural cytotoxicity, purified NK cells were incubated with titrated doses of siplizumab or FcS anti-CD2 mAb in the presence of SPI-801 target cells (HLA).

**Figure 8:**
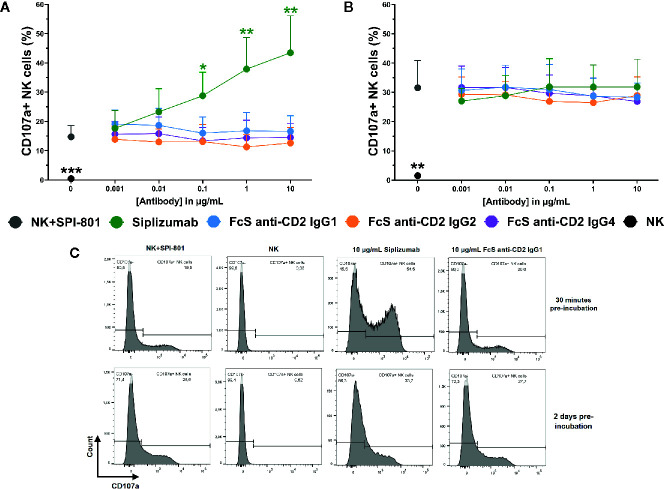
Natural NK cell cytotoxicity. Purified NK cells were pre-incubated without antibody, with 0.001–10 µg/ml Siplizumab or 0.001–10 µg/ml Fc-silent (FcS) anti-CD2 IgG antibodies for 30 min **(A)** or 2 days **(B)** followed by addition of HLA class I^-^ target cells (SPI-801). Significance testing was conducted *via* one-way ANOVA with untreated controls (no antibody) serving as the comparison data set (N=6; *p < 0.05, **p < 0.01, ***p < 0.001). **(C)** Representative histograms of NK cell degranulation in response to HLA class I^-^ target cells without addition of antibody (NK+SPI-801), without addition of antibody and HLA class I^-^ target cells (NK) or in the presence of Siplizumab/FcS anti-CD2 IgG1.

As shown in **Figure 8**, CD2 blockade with FcS anti-CD2 mAb did not significantly inhibit NK cell degranulation in response to SPI-801 target cells, irrespective of whether pre-incubation of NK cells with FcS anti-CD2 mAb lasted 30 min or 2 days prior to target cell addition. After 30 min of pre-incubation, siplizumab significantly increased NK cell degranulation at 0.1–10 µg/ml (p ≤ 0.0482) but not after 2 days of pre-incubation (Repeated-measure one-way ANOVA followed by Dunnett’s multiple comparison test; **Supplementary Tables S16** and **S17**). CD2 blockade did not induce any change in SPI-801 count (data not shown).

### Antibody-Dependent Cytotoxicity

**Figure 9:**
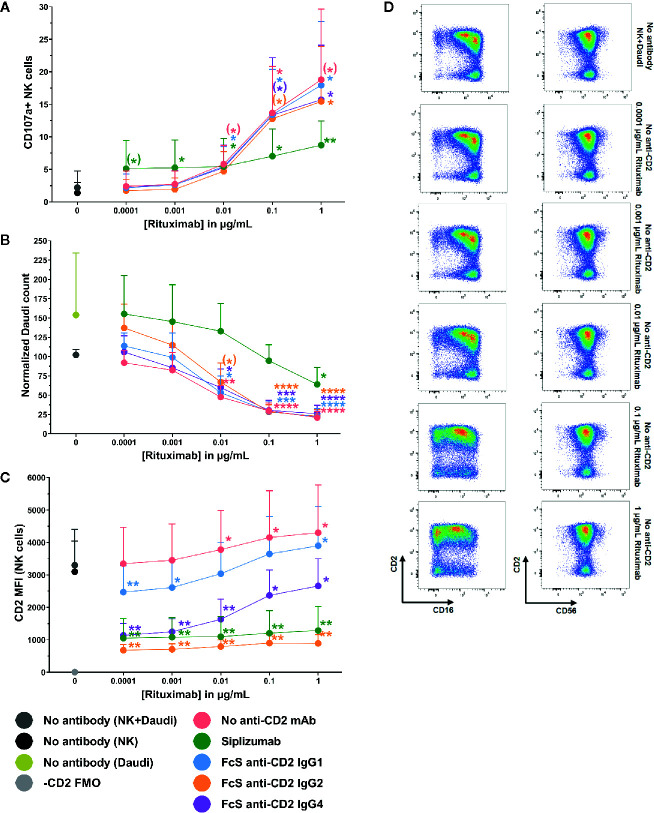
Antibody-dependent cytotoxicity. Purified NK cells were pre-incubated without antibody or with saturating doses of Siplizumab/Fc-silent (FcS) anti-CD2 IgG antibodies before addition of titrated doses of Rituximab and CD20^+^ target cells (Daudi). Significance testing was conducted *via* one-way ANOVA with untreated controls (no antibody) serving as the comparison data set (N=6; ^(^*^)^ p<0.1, *p < 0.05, **p < 0.01, ***p < 0.001, ****p < 0.0001). **(A)** NK cell degranulation. Mean percentage of CD107a^+^ NK cells +SD. **(B)** Target cell depletion. Mean normalized target cell count +SD **(C)** CD2 expression. Average median CD2 fluorescence intensity +SD on NK cells. **(D)** Representative dot plots illustrating increased CD2 expression on NK cells resulting ADCC induction.

To test whether CD2 blockade influences ADCC, purified NK cells were pre-incubated with siplizumab or FcS anti-CD2 mAb before addition of titrated doses of Rituximab and CD20^+^ target cells (Daudi). As shown in **Figures 9A, B**, CD2 blockade with FcS anti-CD2 mAb did not significantly affect NK cell degranulation or target cell killing in response to increasing doses of Rituximab (**Supplementary Tables S18** and** S19**). As displayed in **Figure 9C**, Rituximab induced a significant increase of CD2 expression on NK cells at 0.01–1 µg/ml (p ≤ 0.0248; Repeated-measure one-way ANOVA followed by Dunnett’s multiple comparison test; **Supplementary Table S20**). While anti-CD2 mAbs induced different degrees of CD2 downregulation in this setting, this did not seem to affect ADCC.

## Discussion

The present study has demonstrated that siplizumab can induce NK cell fratricide through ADCC and that CD2 binding by siplizumab does not induce NK cell activation. Further, it was shown that blockade of CD2 with Fc-silent anti-CD2 mAb inhibits CD69 expression on NK cells in MLR. While these results suggested a potential inhibitory effect of CD2 blockade on natural cytotoxicity in MLR, no inhibition of natural cytotoxicity was detected in experiments using purified NK cells. Further, while siplizumab induces ADCC through CD16, this study showed that CD2 blockade by siplizumab does not inhibit ADCC. It should be noted that siplizumab was included in assays evaluating the effect of CD2 blockade on Rituximab-induced ADCC and natural cytotoxicity for completeness, however, NK cell fratricide makes results seen with siplizumab in these assays difficult to interpret.

Limitations of this study included the exclusive use of peripheral blood NK cells which often display different functional profiles and phenotypes when compared to tissue NK cells (18–21). Moreover, all anti-CD2 antibodies used in this study bind the same CD2 epitope [situated near T11.2 and T11.3 in the extracellular domain of CD2 (22)]. Thus, it remains to be determined whether different effects may be induced upon blockade of alternative CD2 epitopes, e.g. in the region binding to LFA3. The data presented in this study had a limited sample size, meaning that it may not necessarily be representative of the average across a broader population.

Previous evidence points towards an important role of CD2 in natural NK cell cytotoxicity. NK cells of patients homozygous for a mutation of L66 in CD16 (L66H) display impaired natural cytotoxicity but normal ADCC (11, 23). This seemed to result from recruitment of CD16 to the NK cell immunological synapse (NKIS) by CD2. Even though IgG antibodies are not involved in natural cytotoxicity, CD16 is recruited to the NKIS, potentially to achieve an enrichment of agonistic receptors in this region. Interestingly, NK cells expressing L66H CD16 displayed lower CD2 expression levels than NK cells expressing wild-type CD16 (11). Additionally, evidence indicates an important role of CD2 in nanotube formation between NK cells and target cells during natural cytotoxicity (24). Further, similar to T cells CD2 seems to mainly assume a peripheral positioning in the NKIS (24) and induces signaling through similar pathways as those observed in T cells (25–27). Surprisingly, no inhibition of natural cytotoxicity resulting from CD2 blockade was detected in this study. This study only used one target cell type (SPI-801) for assessment of natural cytotoxicity and future research should investigate whether CD2 blockade affects natural cytotoxicity against target cells requiring co-expression of CD2 and CD16 for induction of natural cytotoxicity like mel1106.

The relevance of observed CD69 downregulation on NK cells in MLR upon addition of FcS anti-CD2 antibodies remains unclear. While CD2 blockade could not be shown to inhibit ADCC or natural cytotoxicity, CD2-LFA3 interaction may be important for other immunological processes like crosstalk between NK cells and dendritic cells (28) or natural cytotoxicity against target cells requiring co-expression of CD16 and CD2 on effector cells for induction of a cytotoxic response.

Previous clinical trials of CD2-targeting therapies were predominantly conducted with two agents: (1) Alefacept, a fusion protein of an IgG1 Fc fragment and the CD2-binding domain of LFA3 and (2) siplizumab. Both agents are depletory, i.e. their mechanism of action is mediated by both CD2 blockade and FcγR-mediated depletion. Previous studies have detected an initial pro-inflammatory effect of Alefacept treatment in psoriasis patients (29). Recently published evidence indicates no activation effect of siplizumab on T cells in MLR (3) and this study showed that CD2 ligation by siplizumab does not have an inherent activating effect on NK cells. Thus, siplizumab may be a more desirable therapeutic agent for CD2-targeting as it does not exert an agonistic effect upon CD2 binding. Nevertheless, even though CD2 ligation by siplizumab does not induce NK cell activation, siplizumab-mediated ADCC still induces NK cell activation and thus may increase pro-inflammatory signaling by NK cells.

A number of transplantation and autoimmune conditions feature undesired NK cell activation and cytotoxicity. In solid organ transplantation, a large number of cells expressing foreign human leukocyte antigens (HLA) are introduced into the transplant recipient. Mouse models have shown that alloreactive NK cells can be a notable contributor to kidney allograft rejection (30). Further, alloreactive NK cells seemed to contribute allograft rejection in human liver transplantation (31). While this study did not show an inhibition of natural NK cell cytotoxicity by CD2 blockade, depletion of activated NK cells using siplizumab may be a viable treatment modality in conditions where NK cells and T cells are disease-mediating. Addition of siplizumab to commonly used immunosuppressants in transplantation may be desirable since standard immunosuppression inhibits NK cell alloreactivity heterogeneously (32). In autoimmune conditions like rheumatoid arthritis (RA), NK cell count in peripheral blood decreases. In contrast, an increased frequency of NK cells was observed in the synovial fluid of RA patients. Similarly, enrichment of NK cells in tissues affected by autoimmune disease has also been observed in diabetes and psoriasis patients (33, 34). Notably, NK cells in synovial fluid of RA patients are predominantly CD56^bright^, an NK cell subpopulation characterized by high cytokine secretion potential (35) and relatively high CD2 expression levels. Depletion of cells expressing high CD2 levels in autoimmune conditions involving NK cell migration into affected tissues may decrease NK cell tissue infiltration and related cytokine release into tissue microenvironment. Given the high cytokine secretion potential of CD56^bright^ NK cells, depletion of CD2^high^ cells may lower inflammation levels in tissues affected by autoimmune disease.

In addition to ADCC, siplizumab may also induce depletion *via* antibody-dependent cell phagocytosis as a result of CD32 and CD64 binding. Indeed, ADCP-mediated depletion by siplizumab may be underestimated when considering MLR results due the absence of mature phagocytes among PBMC. Interestingly, animal models using other CD2 antibodies (36) or depletory anti-CD20 mAbs (37) have shown accumulation of depleted cells in the liver which contains an abundance of FcγR-bearing cells including hepatic phagocytes.

In conclusion, siplizumab induces NK cell activation and NK cell fratricide through FcγRIIIA-mediated induction of ADCC but not through CD2 ligation. Further, this study found no effect of CD2 blockade by siplizumab on natural cytotoxicity or ADCC induction.

## Data Availability Statement

The raw data supporting the conclusions of this article will be made available by the authors, without undue reservation.

## Author Contributions

CB, FS, and FC carried out experiments and wrote the first manuscript. All authors contributed to the article and approved the submitted version.

## Conflict of Interest

CB, FS, FC, SB, EB and DB are employees of ITB Med AB. SB, EB, and DB own shares in ITB Med AB.

The authors declare that the research was conducted in the absence of any commercial or financial relationships that could be construed as a potential conflict of interest.

## Abbreviations

ADCC, antibody-dependent cell-mediated cytotoxicity; ANOVA, analysis of variance; APC, antigen-presenting cell; DG, deglycosylated; FcγR, Fragment crystallizable gamma receptor; FcS, Fc-silent; KIR, killer cell immunoglobulin-like receptor; NKG2D, natural killer group 2D; HLA, human leukocyte antigen; mAb, monoclonal antibody; NK, natural killer; NKIS, NK cell immunological synapse; LFA3, lymphocyte-associated antigen 3; PBMC, peripheral blood mononuclear cells; RA, rheumatoid arthritis; VPD450, violet proliferation dye 450.
