# Supplementary material for: Siplizumab Induces NK Cell Fratricide Through Antibody-Dependent Cell-Mediated Cytotoxicity
Source: Front Immunol. 2021 Feb 11;12:599526. doi: 10.3389/fimmu.2021.599526 (PMC7904868; doi:10.3389/fimmu.2021.599526)
Supplement: Supplementary file 1 [file DataSheet_1.pdf]

Supplementary information

Supplementary table S1. Cell-based FcγRI signaling assay. Mean normalized luminescence ± SD (N=3).

[ANTIBODY] in nM	Siplizumab		DG siplizumab		FcS anti-CD2 IgG1		FcS anti-CD2 IgG2		FcS anti-CD2 IgG4	
**6.67**	100.0	0.0	125.6	2.7	5.7	0.4	3.8	0.7	3.2	0.4
**1.11**	103.1	6.0	123.0	11.0	5.8	0.9	3.5	0.5	3.0	0.4
**0.19**	106.0	11.9	127.3	8.5	6.0	0.5	3.2	0.3	2.9	0.2
**0.031**	101.7	13.6	107.8	6.9	3.4	0.4	2.9	0.7	2.8	0.2
**0.0051**	26.7	3.9	41.9	2.6	2.5	0.3	2.6	0.1	2.6	0.5
**0.00086**	5.1	0.4	8.5	1.2	2.5	0.3	2.5	0.5	2.9	0.4
**0.000143**	2.4	0.7	2.5	0.4	2.4	0.6	2.4	0.4	2.6	0.5
**0.000024**	2.4	0.5	2.6	0.6	2.7	0.4	2.4	0.3	2.8	0.5

Supplementary table S2. Cell-based FcγRIIA signaling assay. Mean normalized luminescence ± SD (N=3).

[ANTIBODY] in nM	Siplizumab		DG siplizumab		FcS anti-CD2 IgG1		FcS anti-CD2 IgG2		FcS anti-CD2 IgG4	
**666.7**	100.0	0.0								
**111.1**	107.8	17.5								
**18.52**	70.1	3.7								
**3.09**	45.3	1.2								
**0.51**	40.7	4.0								
**0.086**	11.0	1.4								
**0.014**	3.2	0.6								
**0.002**	3.0	0.3								
**66.7**			4.4	0.9	3.1	0.6	6.6	2.0	3.8	0.9
**11.1**			3.2	0.4	3.4	0.8	5.7	0.4	3.1	0.6
**1.85**			3.2	0.4	3.1	0.7	4.4	0.4	3.7	1.4
**0.31**			2.9	0.7	3.0	0.6	4.4	0.5	3.5	0.2
**0.051**			3.4	0.6	2.8	0.6	3.6	0.4	3.3	1.2
**0.009**			2.6	0.4	2.7	0.7	2.9	0.4	2.4	0.2
**0.001**			3.0	0.4	3.0	1.1	3.2	0.1	2.3	0.5
**0.00024**			2.6	0.4	2.5	0.5	3.0	0.5	3.4	0.4

Supplementary table S3. Cell-based FcγRIIIA signaling assay. Mean normalized luminescence ± SD (N=3).

[ANTIBODY] in nM	Siplizumab		DG siplizumab		FcS anti-CD2 IgG1		FcS anti-CD2 IgG2		FcS anti-CD2 IgG4	
**6.67**	100.0	0.0	7.3	0.6	6.8	0.7	3.8	0.5	6.8	0.3
**1.11**	100.0	13.8	8.4	1.9	7.0	0.2	3.2	0.1	7.3	1.1
**0.19**	89.0	7.1	7.0	0.2	7.3	0.9	2.8	0.2	6.7	1.1
**0.031**	74.8	4.4	6.5	0.9	6.4	0.8	4.1	0.4	6.2	0.4
**0.0051**	44.5	3.9	6.5	0.7	6.3	0.5	5.4	0.2	6.4	0.9
**0.00086**	13.4	3.0	6.0	0.9	6.8	1.4	6.2	0.3	6.3	0.1
**0.000143**	6.6	0.9	7.5	2.0	6.7	0.5	6.5	0.6	6.1	1.4
**0.000024**	6.1	0.4	5.9	1.0	6.6	1.5	6.1	0.5	6.4	0.5

Supplementary table S4. CD2 expression. Average CD2 median fluorescent intensity ± SEM (N=5).). Repeated-measure one-way ANOVA results followed by Dunnett's multiple comparison test (* p<0.05, ** p<0.01).

	AVERAGE MFI	SEM
**T CELLS**	651.9	56.3
**CD4 TN**	442.6	7.6
**CD4 TCM**	644.7	48.9
**CD4 TEM**	791.8	102.4
**CD4 TMERA**	468.9	36.6
**CD8 TN**	655.7	28.3
**CD8 TCM**	852.5	147.9
**CD8 TEM**	1165.1	92.7
**CD8 TEMRA**	740.4	117.7
**RTREG**	442.1	43.2
**NSTREG**	621.1	38.7
**ATREG**	777.5	97.0
**B CELLS**	2.7**	21.2
**$CD56^{BRIGHT}$**	713.3	120.6
**$CD56^{DIM}$**	150.5**	33.1
**$CD56^{NEG}$ $CD16^{+}$**	145.1*	46.0

Supplementary table S5. CD2 expression on resting and activated PBMC. Two-tailed paired t-test results (* p<0.05, ** p<0.01). Data shown as average median fluorescence intensity ± SEM.

	T cells		B cells		CD56^{bright}		CD56^{dim}		CD56^{neg} CD16^{+}	
**RESTING**	657.3	102.0	2.7	47.4	713.3	269.6	150.5	74.0	145.1	102.8
**ACTVATED**	11226.6**	3749.1	0.0	196.4	3465.0*	1674.6	990.8**	256.1	990.8**	256.1

Supplementary table S6. CD69+ NK cells in mixed lymphocyte reaction over time. Mean percentage of CD69+ NK cells ± SD (N=12). Repeated-measure two-way ANOVA results followed by Dunnett's multiple comparison test (* p<0.05, ** p<0.01, *** p<0.001, **** p<0.0001).

Time in days	No antibody		Siplizumab		DG siplizumab	
**0**	5.6	4.4				
**1**	14.2	7.9	43.2****	12.4	14.6	6.6
**2**	15.5	6.4	32.7***	10.8	8.1***	4.3
**4**	33.2	19.4	36.5	17.7	16.1**	12.3
**7**	37.8	20.0	36.5	14.6	20.4**	12.7

Supplementary table S7. CD69+ NK cells in autologous lymphocyte culture over time. Mean percentage of CD69+ NK cells ± SD (N=12). Repeated-measure two-way ANOVA results followed by Dunnett's multiple comparison test (* p<0.05, ** p<0.01, *** p<0.001, **** p<0.0001).

Time in days	No antibody		Siplizumab		DG siplizumab	
**0**	5.7	4.4				
**1**	12.0	7.2	43.5****	17.7	15.1*	8.6
**2**	6.3	3.8	34.5****	13.9	7.3	4.5
**4**	9.6	7.7	24.4***	10.7	6.5**	6.3
**7**	15.5	11.1	25.7**	8.2	13.1	8.2

Supplementary table S8. CD69+ NK cells after seven days of mixed lymphocyte reaction incubated with increasing doses of anti-CD2 antibody. Mean percentage of CD69+ NK cells ± SD (N=9). Repeated-measure one-way ANOVA results followed by Dunnett's multiple comparison test (* p<0.05, ** p<0.01, *** p<0.001).

[Antibody] in µg/ml	Siplizumab		DG siplizumab		FcS anti-CD2 IgG1		FcS anti-CD2 IgG2		FcS anti-CD2 IgG4	
**0**	28.6	15.1								
**0.0001**	29.6	15.8	31.3	14.3	34.9	16.3	33.1	14.8	31.3	15.9
**0.001**	28.3	14.9	28.6	13.2	34.2	15.5	29.9	13.7	31.3	14.3
**0.01**	41.0	23.1	23.2	10.9	27.2	14.3	29.4	13.7	28.4	14.6
**0.1**	49.7***	17.7	23.2	11.3	19.8*	10.9	11.9*	4.2	19.9*	10.7
**1**	42.9**	18.5	18.1**	12.0	18.7*	8.1	8.7**	4.3	18.6*	9.9
**10**	42.1*	19.7	14.4**	8.0	14.3***	8.7	8.1**	4.9	14.1**	9.5

Supplementary table S9. CD2 expression on NK cells after seven days of mixed lymphocyte reaction incubated with increasing doses of anti-CD2 antibody. Average median CD2 fluorescence intensity on NK cells ± SD (N=9). Repeated-measure one-way ANOVA results followed by Dunnett's multiple comparison test (* p<0.05, ** p<0.01, *** p<0.001, **** p<0.0001).

[Antibody] in µg/ml	Siplizumab		DG siplizumab		FcS anti-CD2 IgG1		FcS anti-CD2 IgG2		FcS anti-CD2 IgG4	
**0**	3091.0	1074.0								
**0.0001**	2912.0	1193.5	2910.0*	1115.2	3190.3	1082.6	3148.6	1190.1	3013.1	836.9
**0.001**	2783.6*	1043.3	3033.1	1242.4	3146.7	1220.8	3075.2	1145.0	2876.9	968.2
**0.01**	1968.2**	1110.7	2393.1**	919.6	2378.8**	770.4	2447.1**	849.5	2418.6*	630.3
**0.1**	159.8***	61.5	909.3***	405.0	1557.1***	619.1	816.0***	331.7	1245.3***	389.9
**1**	124.8***	51.6	856.3***	439.6	1404.9***	532.5	284.1***	67.3	1059.8***	368.1
**10**	156.2***	86.5	1073.4****	686.5	1466.1***	525.1	288.1***	80.5	950.8***	344.4

Supplementary table S10. NK cell lysis after incubation of purified NK cells with increasing doses of anti-CD2 antibody. Mean percentage of 7-AAD+ NK cells ± SD (N=6). Repeated-measure one-way ANOVA results followed by Dunnett's multiple comparison test (* p<0.05, ** p<0.01, *** p<0.001).

[Antibody] in µg/ml	Siplizumab		FcS anti-CD2 IgG1		FcS anti-CD2 IgG2		FcS anti-CD2 IgG4	
**0**	6.0	2.4						
**0.001**	7.1	2.5	6.7	2.4	6.4	2.3	6.5	2.4
**0.01**	14.6**	3.5	6.6	2.4	6.4	2.5	6.3	2.4
**0.1**	28.8**	6.7	6.6	2.5	6.1	2.3	6.3	2.4
**1**	33.3***	6.4	6.7	2.6	5.6	2.3	6.4	2.4
**10**	35.0***	6.3	6.6	2.7	5.8	2.2	6.3	2.5

Supplementary table S11. Normalized NK cell count after incubation of purified NK cells with increasing doses of anti-CD2 antibody. Mean normalized NK cell count ± SD (N=6). Repeated-measure one-way ANOVA results followed by Dunnett's multiple comparison test (* p<0.05, ** p<0.01, *** p<0.001, **** p<0.0001).

[Antibody] in µg/ml	Siplizumab		FcS anti-CD2 IgG1		FcS anti-CD2 IgG2		FcS anti-CD2 IgG4	
**0**	100.0	0.0						
**0.001**	103.1	3.0	105.7	6.1	90.0	14.3	98.7	10.1
**0.01**	81.0*	11.3	105.3	7.3	97.3	10.1	104.3*	2.3
**0.1**	47.8***	10.6	102.0	15.1	100.1	6.8	106.6	4.6
**1**	41.0****	8.4	105.5	10.9	104.7	4.6	106.3	5.6
**10**	41.1****	8.2	107.4	8.2	103.2	7.3	102.6	14.3

Supplementary table S12. Normalized CD56^{dim} NK cell count after incubation of purified NK cells with increasing doses of anti-CD2 antibody. Mean normalized CD56^{dim} NK cell count ± SD (N=6). Repeated-measure one-way ANOVA results followed by Dunnett's multiple comparison test (* p<0.05, ** p<0.01, *** p<0.001, **** p<0.0001).

[Antibody] in µg/ml	Siplizumab		FcS anti-CD2 IgG1		FcS anti-CD2 IgG2		FcS anti-CD2 IgG4	
**0**	100.0	0.0						
**0.001**	102.8	2.8	106.1	6.1	90.1	14.2	99.0	9.8
**0.01**	81.5*	9.8	105.7	6.9	97.8	10.5	104.4*	2.8
**0.1**	50.7***	10.7	102.4	14.5	101.9	8.2	106.9*	4.4
**1**	43.8****	8.6	106.3	10.3	106.3	4.8	106.9	5.3
**10**	44.0****	8.4	107.2	8.1	104.9	8.0	102.5	14.1

Supplementary table S13. Normalized CD56^{bright} NK cell count after incubation of purified NK cells with increasing doses of anti-CD2 antibody. Mean normalized CD56^{bright} NK cell count ± SD (N=6). Repeated-measure one-way ANOVA results followed by Dunnett's multiple comparison test (* p<0.05, ** p<0.01, *** p<0.001, **** p<0.0001).

[Antibody] in µg/ml	Siplizumab		FcS anti-CD2 IgG1		FcS anti-CD2 IgG2		FcS anti-CD2 IgG4	
**0**	100.0	0.0						
**0.001**	94.6	10.2	95.4	13.7	89.2	14.5	91.4	13.4
**0.01**	42.0**	25.8	98.5	22.3	83.6	13.5	102.4	9.8
**0.1**	10.2****	5.1	93.9	22.2	64.7**	11.0	103.6	18.3
**1**	7.0****	2.3	96.1	27.8	64.2**	14.8	100.1	19.9
**10**	7.3****	3.3	108.9	12.6	71.9*	13.6	103.3	19.7

Supplementary table S14. CD107a expression on NK cells after incubation of purified NK cells with increasing doses of anti-CD2 antibody. Mean percentage of CD107a+ NK cells ± SD (N=6). Repeated-measure one-way ANOVA results followed by Dunnett's multiple comparison test (* p<0.05, ** p<0.01, *** p<0.001).

[Antibody] in µg/ml	Siplizumab		FcS anti-CD2 IgG1		FcS anti-CD2 IgG2		FcS anti-CD2 IgG4	
**0**	1.6	1.9						
**0.001**	2.0*	1.9	1.9	2.4	1.9	2.2	1.7	2.0
**0.01**	4.3**	2.4	1.7	2.1	2.0	2.2	1.7	1.9
**0.1**	10.9**	4.8	1.7	2.0	2.1	2.2	1.7	1.9
**1**	13.0**	4.9	1.6	1.7	2.1	2.4	1.5	1.8
**10**	14.8***	4.4	1.5	1.7	2.0	2.2	1.7	1.9

Supplementary table S15. CD16 expression on NK cells after incubation of purified NK cells with increasing doses of anti-CD2 antibody. Average median CD16 fluorescence intensity on NK cells ± SD (N=6). Repeated-measure one-way ANOVA results followed by Dunnett's multiple comparison test (* p<0.05, ** p<0.01, *** p<0.001, **** p<0.0001).

[Antibody] in µg/ml	Siplizumab		FcS anti-CD2 IgG1		FcS anti-CD2 IgG2		FcS anti-CD2 IgG4	
**0**	1673.8	546.3						
**0.001**	1577.5	594.2	1645.5	612.3	1624.0	539.9	1598.7	602.7
**0.01**	553.7**	344.3	1671.2	636.6	1577.8	601.4	1612.3	612.1
**0.1**	118.9**	105.5	1624.7	589.7	1491.0*	593.2	1589.5	592.9
**1**	80.3**	52.6	1599.2	557.4	1464.2*	620.0	1589.2	565.5
**10**	69.3**	37.0	1677.5	594.5	1502.8	609.4	1602.5	585.4

Supplementary table S16. CD107a expression on NK cells after incubation of purified NK cells with increasing doses of anti-CD2 antibody and SPI-801 target cells. Mean percentage of CD107a+ NK cells ± SD (N=6). Pre-incubation of NK cells with anti-CD2 mAbs for 30 minutes before target cell addition. Repeated-measure one-way ANOVA results followed by Dunnett's multiple comparison test (* p<0.05, ** p<0.01, *** p<0.001).

[Antibody] in µg/ml	Siplizumab		FcS anti-CD2 IgG1		FcS anti-CD2 IgG2		FcS anti-CD2 IgG4		NK only	
**0**	14.7***	3.8							0.4***	0.2
**0.001**	17.6	6.1	19.1	4.8	13.9	4.5	15.7	4.2		
**0.01**	23.3	7.9	18.6	5.8	13.0	4.4	15.8	5.7		
**0.1**	28.8*	8.0	16.0	5.5	13.1	5.9	13.4	4.5		
**1**	37.9**	10.9	16.8	6.2	11.3	4.2	14.4	6.0		
**10**	43.5**	12.6	16.6	5.3	12.6	5.3	14.5	4.7		

Supplementary table S17. CD107a expression on NK cells after incubation of purified NK cells with increasing doses of anti-CD2 antibody and SPI-801 target cells. Mean percentage of CD107a+ NK cells ± SD (N=6). Pre-incubation of NK cells with anti-CD2 mAbs for 48 hours before target cell addition. Repeated-measure one-way ANOVA results followed by Dunnett's multiple comparison test (* p<0.05, ** p<0.01, *** p<0.001).

[Antibody] in µg/ml	Siplizumab		FcS anti-CD2 IgG1		FcS anti-CD2 IgG2		FcS anti-CD2 IgG4		NK only	
**0**	31.6	9.3							1.5**	0.6
**0.001**	27.0	4.4	30.7	7.4	29.3	5.9	31.7	7.3		
**0.01**	28.8	6.9	31.8	7.5	29.1	4.5	31.6	6.9		
**0.1**	31.9	9.6	31.0	8.5	26.9	5.1	29.7	6.2		
**1**	31.8	7.5	28.7	6.1	26.5	4.4	28.8	6.1		
**10**	31.9	9.4	28.3	4.8	28.9	6.4	26.9	5.0		

Supplementary table S18. CD107a expression on NK cells after incubation of purified NK cells with increasing doses of Rituximab and CD20^{+} target cells. Mean percentage of CD107a+ NK cells ± SD (N=6). Repeated-measure one-way ANOVA results followed by Dunnett's multiple comparison test ($^{(*)}$ p<0.1, * p<0.05, ** p<0.01, *** p<0.001).

[Rituximab] in µg/ml	No anti-CD2		Siplizumab		FcS anti-CD2 IgG1		FcS anti-CD2 IgG2		FcS anti-CD2 IgG4	
**0**	2.2	2.5								
**0.0001**	2.4	2.4	5.1$^{(*)}$	4.3	2.2	2.1	1.7	1.7	2.3	2.7
**0.001**	2.7	2.0	5.3*	4.3	2.6	2.2	1.9	1.8	2.6	2.2
**0.01**	5.8$^{(*)}$	2.8	5.4*	4.3	5.5*	3.0	4.7	3.0	5.3	3.4
**0.1**	13.7*	7.1	7.0*	4.2	13.4*	7.0	12.8$^{(*)}$	8.0	13.3$^{(*)}$	8.8
**1**	18.8$^{(*)}$	10.9	8.7**	3.7	17.9*	9.8	15.4*	8.5	15.7*	8.4

Supplementary table S19. Normalized target cell count after incubation of purified NK cells with increasing doses of Rituximab and CD20^{+} target cells. Mean normalized target cell count ± SD (N=6). Repeated-measure one-way ANOVA results followed by Dunnett's multiple comparison test ($^{(*)}$ p<0.1, * p<0.05, ** p<0.01, *** p<0.001, **** p<0.0001).

[Rituximab] in µg/ml	No anti-CD2		Siplizumab		FcS anti-CD2 IgG1		FcS anti-CD2 IgG2		FcS anti-CD2 IgG4	
**0**	100	0								
**0.0001**	91.9	16.2	155.3	49.8	113.9	16.7	137.1	30.8	106.0	21.1
**0.001**	82.4	22.8	145.3	47.9	99.0	31.4	114.9	32.1	85.5	26.5
**0.01**	47.5**	15.2	132.9	35.9	53.2*	21.6	67.0$^{(*)}$	24.6	59.9*	24.0
**0.1**	29.6****	10.3	94.7	20.7	29.0***	13.3	28.2****	9.5	30.3***	13.1
**1**	21.0****	8.0	64.1*	22.1	22.1****	10.6	22.8****	8.4	26.0****	10.8

Supplementary table S20. CD2 expression on NK cells after incubation of purified NK cells with increasing doses of Rituximab and CD20^{+} target cells. Median CD2 fluorescence intensity on NK cells ± SEM (N=6). Repeated-measure one-way ANOVA results followed by Dunnett's multiple comparison test (* p<0.05, ** p<0.01, *** p<0.001).

[Rituximab] in µg/ml	No anti-CD2		Siplizumab		FcS anti-CD2 IgG1		FcS anti-CD2 IgG2		FcS anti-CD2 IgG4	
**0**	3299.7	1105.6								
**0.0001**	3346.0	1118.4	1050.0**	603.1	2469.3**	816.0	679.2**	175.8	1136.7**	369.9
**0.001**	3452.2	1118.3	1082.0**	602.4	2610.5*	819.7	706.5**	169.7	1249.7**	397.0
**0.01**	3780.0*	1204.6	1098.8**	614.6	3037.2	966.1	792.5**	206.8	1632.5**	618.7
**0.1**	4155.3*	1440.2	1206.5**	690.8	3647.2	1153.5	898.2**	258.2	2369.7*	776.7
**1**	4303.0*	1473.3	1287.3**	743.2	3898.5*	1212.8	889.3**	267.4	2660.2*	841.5
